# Supplementary figures and images for: Production of IFN-γ by splenic dendritic cells during innate immune responses against Francisella tularensis LVS depends on MyD88, but not TLR2, TLR4, or TLR9
Source: PLoS One. 2020 Aug 3;15(8):e0237034. doi: 10.1371/journal.pone.0237034 (PMC7398525; doi:10.1371/journal.pone.0237034)

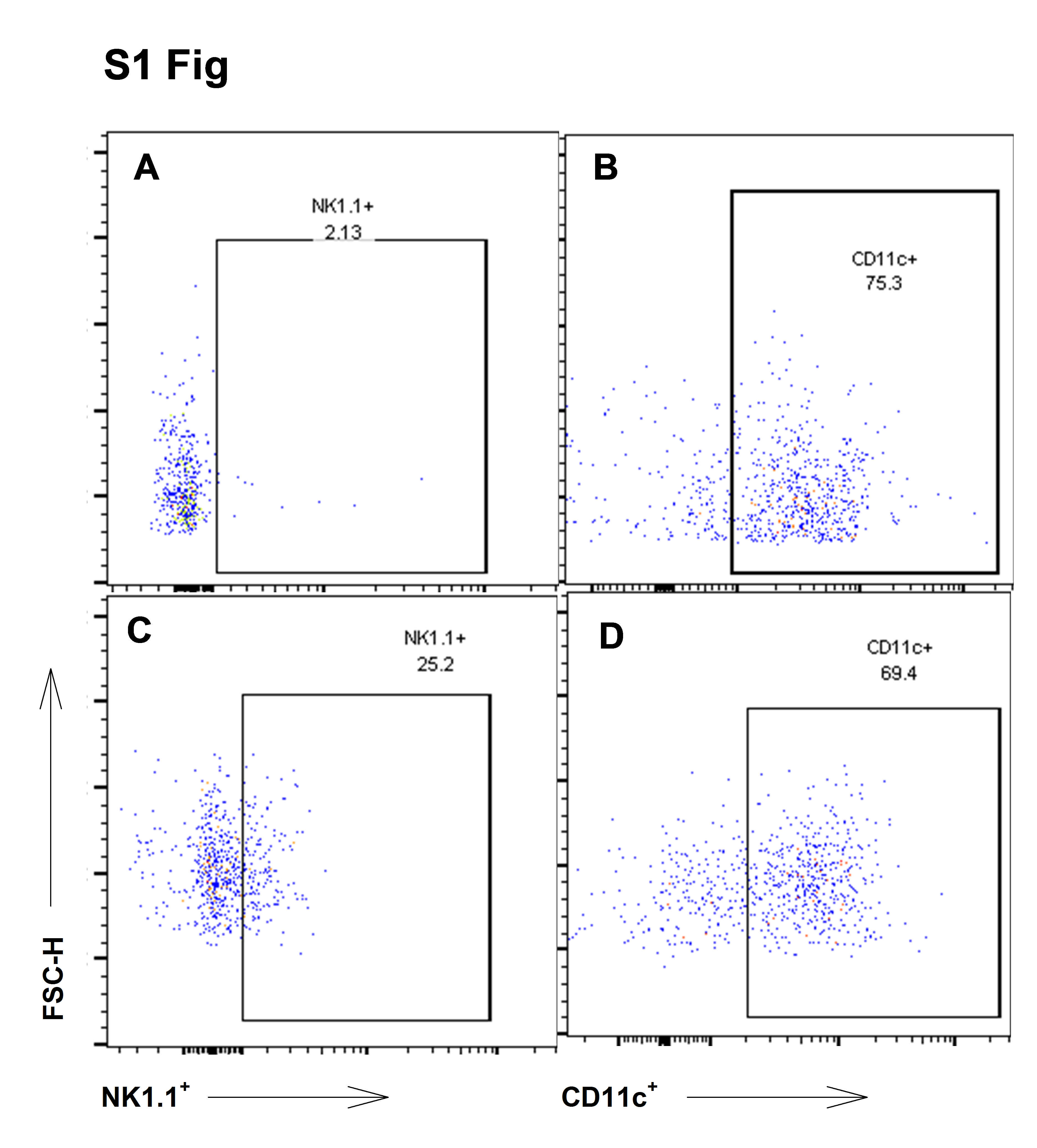

Supplement: S1 Fig — Rag1 KO mice were depleted in vivo of NK1.1+ cells and then infected with 105 LVS i.d, Splenocytes derived from these mice were enriched in vitro for CD11c+ cells using magnetic beads, and the resulting cells were analyzed by flow cytometry. After exclusion of fragments, aggregates and dead cells, CD45+ cells were gated for NK1.1+ cells (Panel A). Alternatively, CD45+ cells were excluded of Gr1+ CD11b+ cells and then gated for CD11c+ cells (Panel B). Panels C and D show total splenocytes from Rag1 KO, not depleted of NK and not purified of CD11c. (TIF) [file pone.0237034.s001.tif]

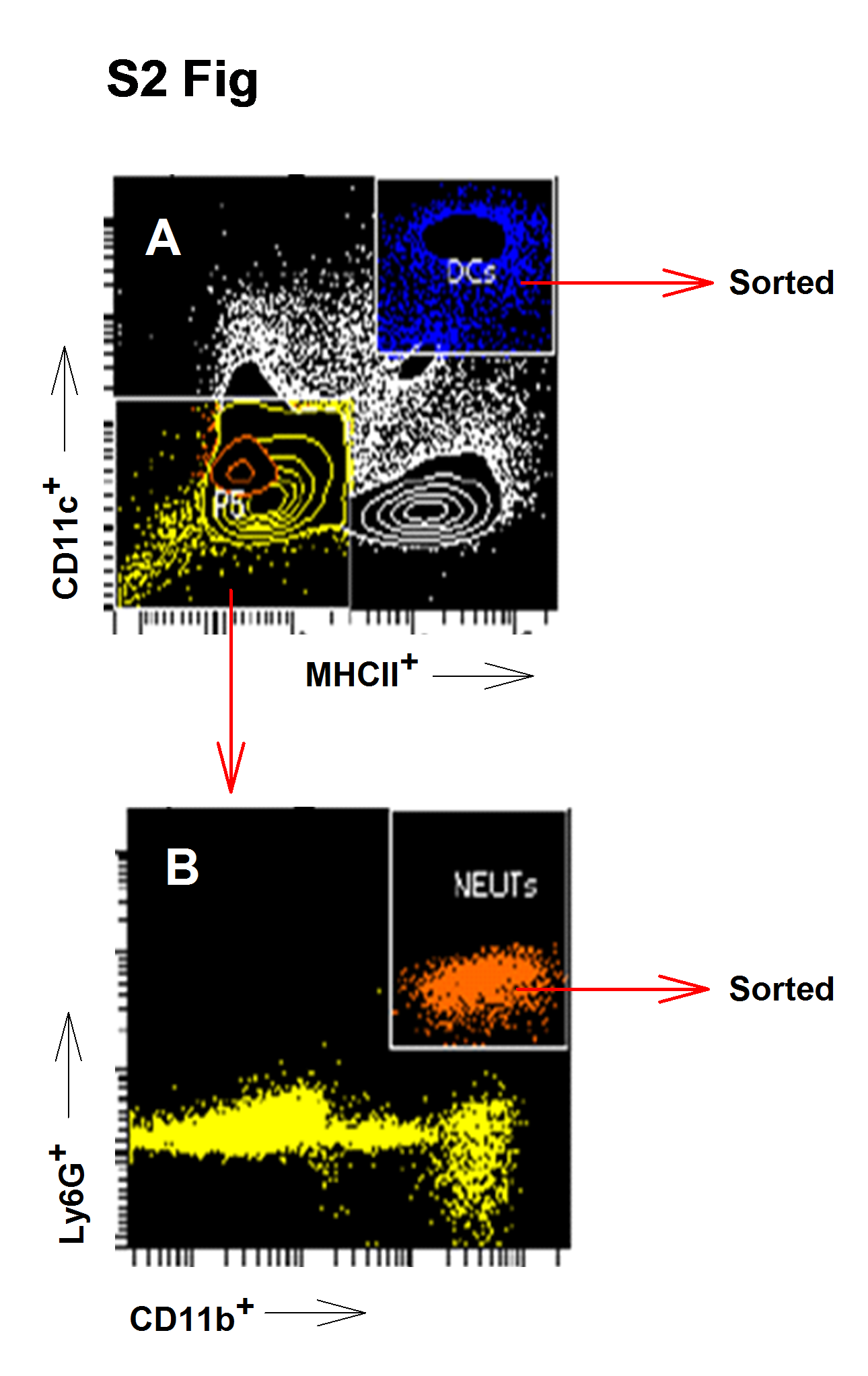

Supplement: S2 Fig — C57BL/6 mice were infected with 105 LVS i.d. Splenocytes from naïve and LVS-infected mice were depleted of B and T cells by magnetic beads and stained for flow cytometry. After exclusion of fragments, aggregates, and dead cells, conventional DC were sorted using CD11c and MHCII markers and cells within the upper right blue quadrant collected (A). To sort neutrophils, CD11c- MHCII- cells were subsequently gated for CD11b+ Ly6G+ and cells within the upper right red quadrant were collected (B). RNA and DNA were purified from sorted cells and used for qRT-PCR (see Table 1). Data are from one independent experiment representative of three independent experiments of similar design and outcome. A similar strategy was used to sort cells from KO mice. (TIF) [file pone.0237034.s002.tif]

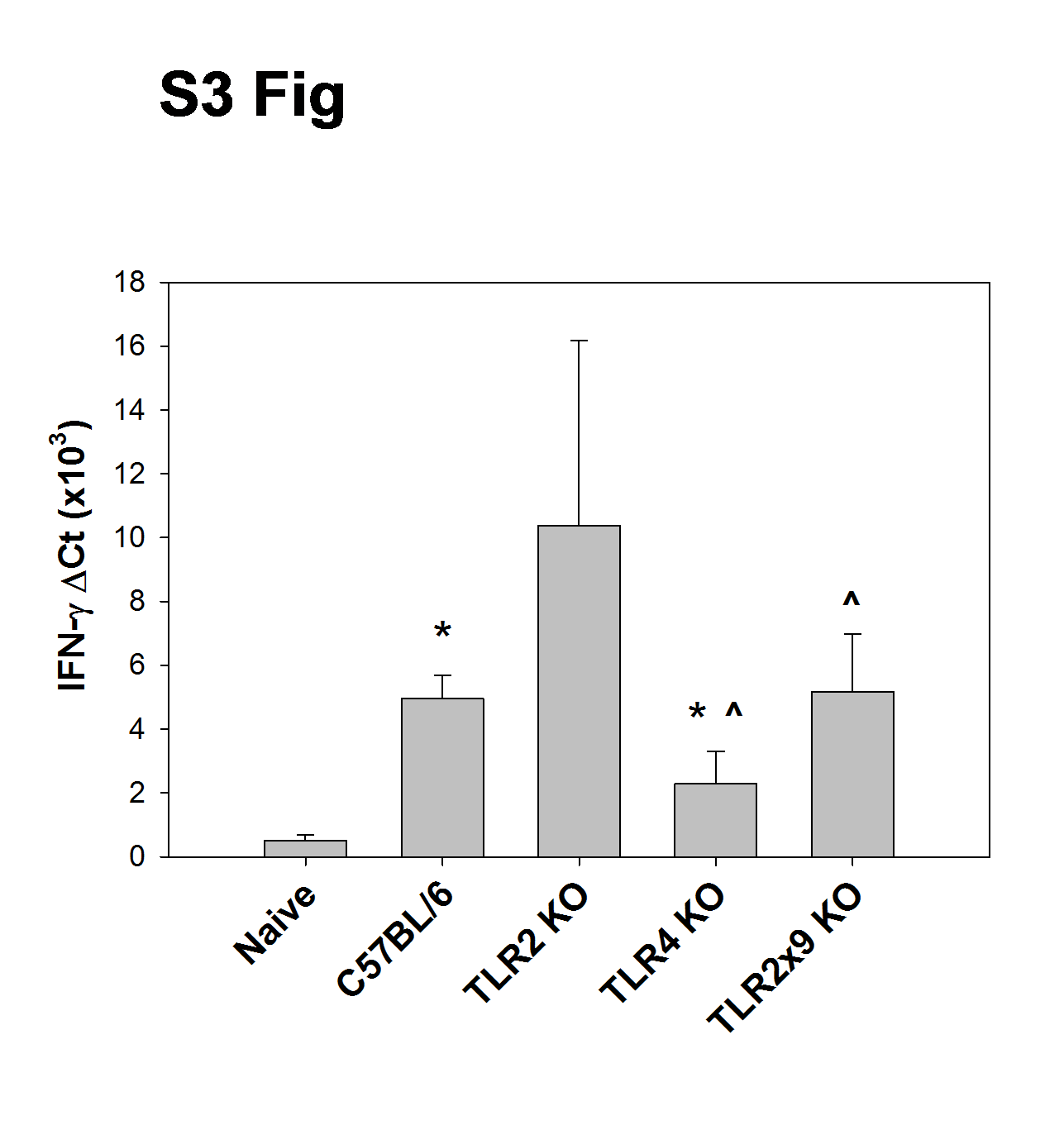

Supplement: S3 Fig — The indicated mice were infected with 105 LVS i.d. After four days, mice were euthanized and gene expression of IFN-γ was determined from the harvested splenocytes by qRT-PCR. Values shown are the mean Δct ± SD derived from three individual mice, multiplied by 1000 for ease of presentation. * and ^ indicate significant differences (P < 0.05) between groups. (TIF) [file pone.0237034.s003.tif]
